# Current Status and Prospects of Polymer Powder 3D Printing Technologies

**DOI:** 10.3390/ma13102406

**Published:** 2020-05-23

**Authors:** Yue Wang, Zhiyao Xu, Dingdi Wu, Jiaming Bai

**Affiliations:** Shenzhen Key Laboratory for Additive Manufacturing of High performance Materials, Department of Mechanical and Energy Engineering, Southern University of Science and Technology, Shenzhen 518055, China; 11950015@mail.sustech.edu.cn (Y.W.); xuzy@mail.sustech.edu.cn (Z.X.); 11749085@mail.sustech.edu.cn (D.W.)

**Keywords:** polymer powder, 3D printing, binder jetting, selective laser sintering, high-speed sintering

## Abstract

3D printing technology, which greatly simplifies the manufacturing of complex parts by a two-dimensional layer-upon-layer process, has flourished in recent years. As one of the most advanced technology, polymer powder 3D printing has many advantages such as high materials utilization rate, free of support structure, great design freedom, and large available materials, which has shown great potential and prospects in various industry applications. With the launch of the Multi jet Fusion system from HP, polymer powder 3D printing has been attracting more attention from industries and researchers. In this work, a comprehensive review of the main polymer powder-based 3D printing methods including binder jetting, selective laser sintering, high-speed sintering were carried out. Their forming mechanism, advantages and drawbacks, materials, and developments were presented, compared, and discussed respectively. In addition, this paper also gives suggestions on the process selection by comparing typical equipment parameters and features of each technology.

## 1. Introduction

3D printing, also known as “additive manufacturing” (AM), is an emerging technology attracting interests from industries and academic communities in recent years [1,2,3]. It is a novel manufacturing process which by joining materials layer by layer to build three-dimensional parts from digital model files directly. AM is considered as one of the key technologies for transforming traditional manufacturing to intelligent manufacturing which aims to take advantage of advanced information and manufacturing technologies to produce products. It is capable of producing parts from micro to macro scale, the precision and accuracy of the parts are related to the specific techniques and printing parameters [4,5,6].

Great design freedom, materials saving, customization, and the ability to manufacture complex geometry with high precision are the main advantages of 3D printing [7]. Therefore, AM has promising application prospects in the fields of prototyping, aerospace, machinery manufacturing, and art ware production [8,9]. With the deepening of research, 3D printing has been extended to more fields. For example, in construction industry, there were a group of houses in China being successfully mass printed in less than a day [10]. Besides, this technology has been regarded as an effective solution to treat tissue defects in biomedical field since 3D printing can produce a variety of medical implants and scaffolds [11]. 

**Figure 1:**
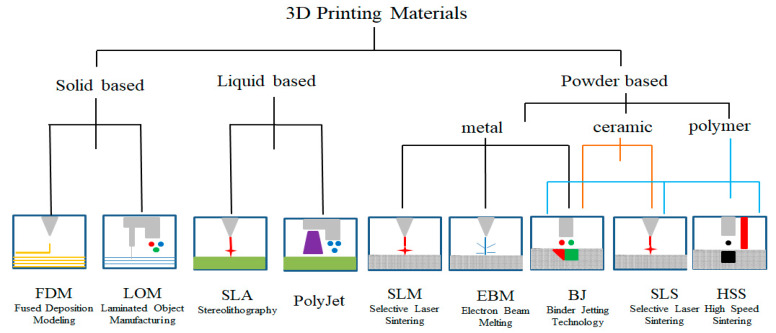
3D printing technical classification according to printing materials.

Based on the ASTM standard, AM processes are classified into seven categories: material extrusion, powder bed fusion, vat photo polymerization, binder jetting, sheet lamination, directed energy deposition and material jetting [12,13,14]. Based on different forms of input materials, 3D printing technology can be divided into solid, liquid, and powder based. For solid based, plastic wire material and layers of adhesive-coated paper, plastic, or metal laminates can be processed to fabricate 3D parts in FDM and LOM respectively. Photosensitive resin is the common raw material for liquid-based technologies. Powder-based materials can be divided into three types: metal powder, ceramic powder, and polymer powder. The classification is shown in Figure 1.

Powder materials have great advantages of great utilization rate, wide materials range, and easy manufacturing process [15]. Meanwhile, the excessive powder is removed at the end of the building circle and can be reused in the next printing process. For polymer powder materials, powders can act as support for the part, allowing more design freedom, which is a big challenge for other 3D printing technologies.

**Figure 2:**
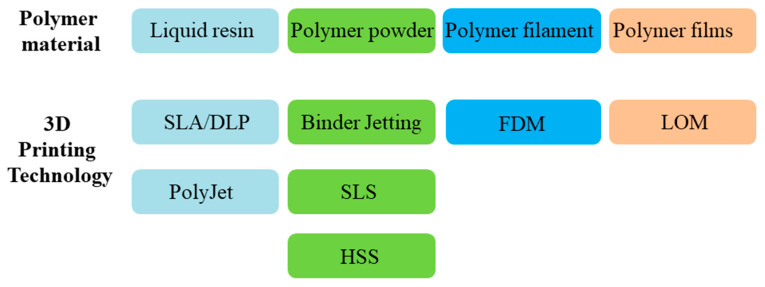
Polymer materials used with specific building methods in additive manufacturing (AM).

Polymers are by far the most common materials for 3D printing technology [16], and their applications are diverse in different fields. Unparalleled by metals and ceramics, polymer-based 3D printing plays an important role in the multifunctional and multi-material systems including living biological systems as well as life-like synthetic systems, especially some degradable polymers, such as polylactide (PLA) and polyglutamic (PGA) [16,17]. The range of polymers used in AM includes liquid resin, polymer powder, polymer filament, and polymer films; the connections between polymers and technologies are shown in Figure 2. 

Since 1980s, polymer powder based 3D printing technologies has prospered because of its dual advantages of powder properties and polymer applications. However, there are many limitations in the polymer powder-based 3D printing process. For example, anisotropic behavior that shows different properties in their different directions has a big influence on the quality of the produced part. Poor surface appearance and mechanical properties, limited materials and high cost are also crucial problems that pose challenges in utilizing this technology in various needs. 

**Figure 3:**
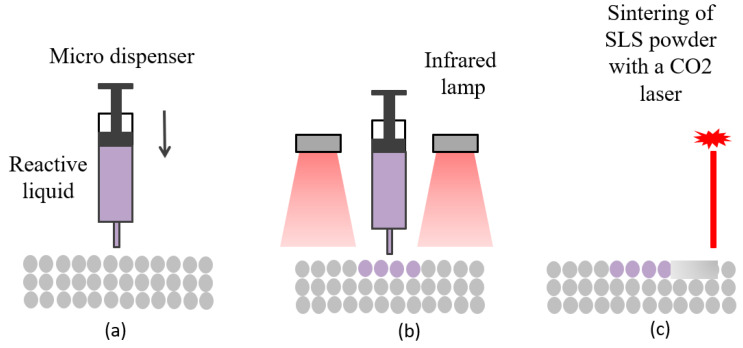
Scheme of the hybrid selective laser sintering (SLS) process. (**a**) Fluid material is injected to SLS powder bed; (**b**) curing reaction of the liquid initiated via IR radiation; (**c**) SLS powders are melted with a CO_2_ laser.

Ongoing studies about polymer powder-based AM technologies that aim to study the forming mechanism and develop the powder materials, process, and devices are conducted nowadays. Sutton et al. [18] reviewed the powder characterization techniques and studied the connections between powder characteristics and part properties in powder bed fusion 3D printing process. Dizon et al. [19] summarized the mechanical test standards for 3D printed parts and reported the properties under different loading types such as tensile, compressive, fatigue, bending, impact, and others, the effects of fillers and post-processing procedure on the mechanical properties of produced parts have also been discussed. For materials, because of the limited mechanical properties and functionalities of printed pure polymer parts, there is a crucial need to develop printable polymer composites with high performances [20]. Incorporation of particle, fiber, or nanomaterial reinforcements into polymer can achieve composites that have high mechanical performance and excellent functionality. Some researchers studied new hybrid AM technique to overcome the problems of manufacturing multi-material parts in one additive process. For example, Katrin et al. [21] introduced a new AM process that combines reactive liquid and polymer powder (PEBA) to generate multi-material selective laser sintering (SLS) parts; it has the potential to create new properties in SLS process, as shown in Figure 3. In the meantime, commercial powder-based printing devices have also been increasingly developed in recent years. EOS launched a P810 system specially designed to deal with HT-23 (reinforced carbon fiber PEKK) materials in the aerospace industry in 2018. 

With the launch of the Multijet Fusion system from HP, polymer powder 3D printing has attracted more attention from industries and users. Currently, typical polymer powder 3D printing technologies include: binder jetting technology (BJ), selective laser sintering (SLS), and high-speed sintering (HSS).This paper introduces their working principles, characteristics, developments, research focus, and prospects, and gives suggestions on the process selection by comparing typical equipment parameters and features of each technology.

## 2. Binder Jetting Technology (BJ)

### 2.1. Overview

**Figure 4:**
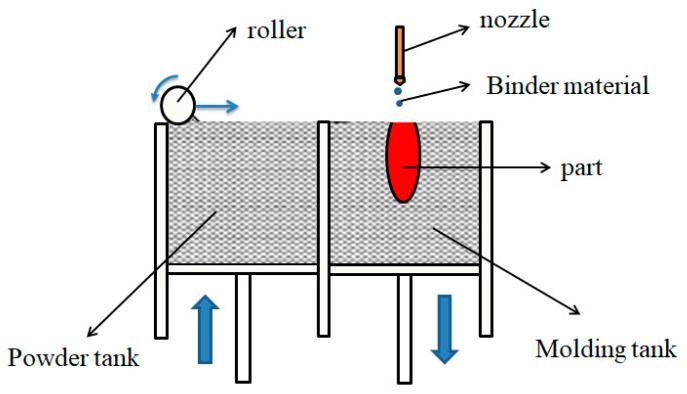
Working scheme of binder jetting technology (BJ).

Binder jetting Technology is a process that fabricates parts by depositing liquid binder to the selective areas of powder material. To be specific, a digital model is sliced to generate cross-section data at the beginning. After the roller lays the powder in the powder tank on the working platform, the nozzle is controlled according to the data to deposit liquid binder selectively on the powder surface to complete one printing process. The molding tank is lowered by one layer thickness, and the powder tank is raised by one layer thickness for the next printing. Layer stacking until a part is finished. Because the parts bonded with the binder materials are low in strength, a certain post-processing is usually required to obtain the final part. Therefore, a successful realization of this process involves not only the printing process itself, but also a suitable post-processing procedure, both of them have a big influence on determining the mechanical performances of the part produced [22]. The BJ working scheme is shown in Figure 4.

**Figure 5:**
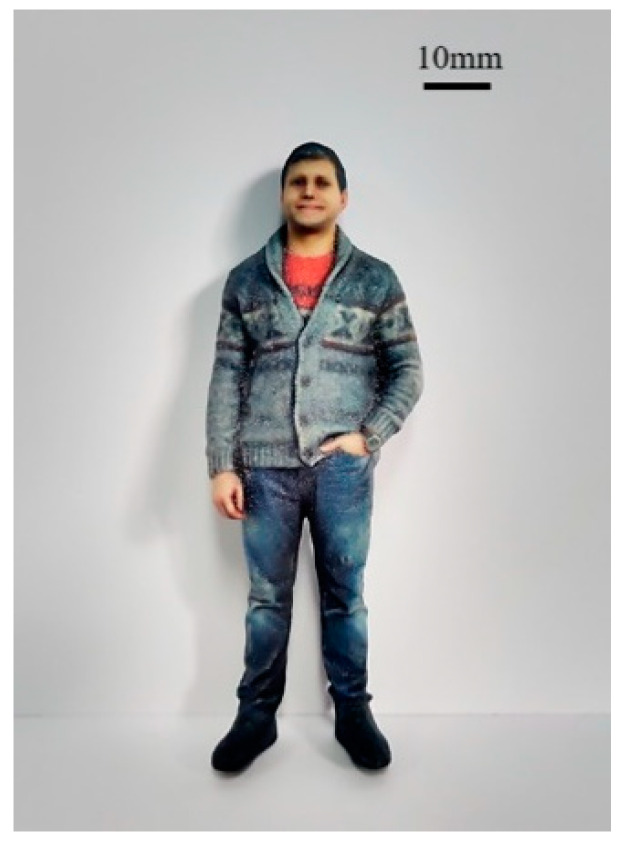
BJ printed full-color portrait.

By analyzing the working principle of BJ, the technical characteristics can be summarized. First of all, powder material in this process can be selected flexibly including just about anything that is available as a spreadable powder with a suitable particle size. Second, the nozzle in BJ can perform array scanning instead of laser spot scanning, so the printing speed is fast, which is beneficial when printing of large-sized parts. Third, by applying different colored inks, BJ can realize colorful printing with multiple colors within individual layers and are thus popular for the fabrication of 3D multicolored models for 3D visualization. Finally, room temperature processing environment is enough for BJ. Furthermore, the mechanics and process of BJ are relatively simple and complicated parts can also be completed. However, the technology is essentially a physical bond process, the internal bonding force is weak and porous, so the printed parts are weak in strength and the surface roughness is poor. Therefore, this technology is mostly used to make conceptual models [23,24], as demonstrated in Figure 5.

### 2.2. The Development of BJ

BJ was originally proposed by Emanual Sachs who was from Massachusetts Institute of Technology and applied for the patent in 1989 [25]. In 1995, MIT licensed this technology to Z Corporation, which has launched a series of BJ printers since 1997. In 2012, Z Corporation was acquired by 3D Systems, and in combination with the traditional selective laser sintering technology, the Z series BJ printers were released. In 2014, Dutch engineer developed the first open source BJ device named Plan B which used standard electronic circuits and existing inkjet components and the data was free for everyone [26]. Since the 21st century, the research on this technology has experienced from a single material to composites, from single-nozzle line scanning printing to multi-nozzle area scanning printing, and has become increasingly competitive in printing speed, precision, strength, and application. Nowadays, the more mature BJ equipment mainly includes some Pro jet series products of 3D Systems and VX series products of Voxeljet [27,28]. Since the emergence of color printers, BJ has developed more rapidly and the number of equipment sales has increased rapidly.

### 2.3. Research Focus and Prospects of BJ

The current research focus of BJ is mainly on hardware, materials, and process optimization.

#### 2.3.1. Hardware

Nozzle is the core hardware of a BJ printer. The performance of the nozzle directly determines the quality of the final product. At present, most of the nozzles on the market are thermal bubbles or piezoelectric commercial nozzles. The thermal bubble nozzle sprays droplets by heating nozzle to generate bubbles, which forces droplets out to the parts. Because of the working condition of high temperature and high pressure, the nozzle can be corroded and blocked easily. As ink is jetted through the bubble, the directivity and volumes of the ink are not well controlled, which inevitably affects the mechanical properties of the parts while the printing speed of this nozzle can be better [29]. The piezoelectric nozzle sprays the ink by applying a voltage to the piezoelectric crystal to cause a slight deformation. So this way has lots of advantages. For example, shape, size of the droplets, and jetting speed can be controlled [30]. Nowadays, the main nozzle manufactures are HP, Epson, Ricoh, Toshiba, and Xaar.

The nozzle has strict requirements on the viscosity, surface tension, working temperature of the sprayed liquid, so the available binder materials are limited at the moment, which affects the development of BJ. In the meantime, the printing resolution and speed can be influenced by the nozzle as well. In recent years, nozzle manufacturers have continued to use new technologies to enhance printing performances, such as circulating ink technology, nozzle redundancy technology, and precise positioning technology. However, nozzle problems are still a big challenge for BJ machines and devices. Therefore, a high-performance nozzle is essential for BJ industries for further development and better applied.

#### 2.3.2. Printing Materials

At present, there are many kinds of printing materials that can be used for BJ technology, especially in the field of mold forming, such as ceramics, metals, sand powder, and so on. Nylon and gypsum powder also have excellent application value in the manufacture of prototype parts. At the same time, BJ technology can be widely used in the biomedical field, forming tablets and some special functional powders and scaffolds. In the micro-nano manufacturing fields, some metals can be used to manufacture various semiconductor conventional materials [31,32,33,34,35,36]. Although there are many kinds of printing materials available for BJ, it is still a research focus of this technology to continuously develop printing materials. A large percentage of BJ materials developed are still metals and ceramics. AlCoCrFeNi alloy, copper foam structures, alumina nitride components were fabricated by BJ technologies recently for different applications [37,38,39]. Limited studies have been done with BJ for pure polymers while a few work has been done for polymer composites. Kim et al. [40] developed ceramic/polymer composites with a lightweight structure by BJ 3D printing process. A large amount of the polymers such as polylactic acid (PLA), polycraprolactone(PCL), collagen are used in BJ are applied into biomedical applications [41]. For example, Inzana et al. [42] printed calcium phosphate and collagen composites scaffolds for bone regeneration. With the advancement of BJ technology, there will be more advanced composites appearing, which will have improved mechanical properties and multiple functions.

In the powder formulation section, additives and fillers can be added to the printing materials in order to improve the powder despositability, printing behavior, and part quality. For example, long fibers can be added to the powder to reinforce the final part, while shorter fibers can increase dimensional stability [43]. Besides, adding cellulose can enhance the strength of powder bed; adding lecithin can improve the dimensional accuracy [44]. For printing materials, powder characteristics are also a crucial factor. Fine powders have the potential to get increased surface roughness, and high accuracy [45]. Powder with smaller mean particle size resulted in better surface finish and higher final densities. The hardness produced also exhibited higher values compared to the parts fabricated with the larger particles [46]. Powders with high sphericity tend to flow well and have low internal friction as well [47].

#### 2.3.3. Binder Materials

Liquid binder has a key role in the forming process of BJ since their physical properties, amount, and saturation can influence the quality of the printed components [48,49,50,51]. The excessive binders would result in dimensional inaccuracies; insufficient amount would decrease the mechanical properties. Miyanaji et al. [52] have developed a physics-based model to predict the optimal saturation amount that ensures the surface finish and structural integrity. Enneti et al. [53] studied effects of binder saturation and powder layer thickness on the strength of the green parts. The studies showed the strength of the parts will increase when binder saturation increases for a given layer thickness. Besides, for a given binder saturation, the optimal layer thickness is also different. The results give some references when choosing layer thickness and binder saturation.

Binder solution can act by different mechanisms. The first is self-binding, such as silicone adhesive. The second is that binder can trigger a reaction between powders to bond them together, such as deionized water. The third is that there will be reactions between itself and powder to achieve the bond, such as the acid calcium sulfate used for alumina powder [44,54,55].

As different types of binder materials are used in different printing materials, BJ technology has more and more higher requirements on the binders. So many scholars are devoted to improving the performance of the existing binder materials and develop new materials. For example, Huang and Ye [56] used modified silicate as binders to investigate the effects of process parameters in order to find the optimum conditions. Some researchers also did some studies on the simulation of binder materials aims to improve the forming precision and forming efficiency. Yang [57] studied the impact and infiltration process of binder by the combination of simulation and experiment, and analyzed the influence of different parameters on the results. It can be concluded that the larger Reynolds number, the larger the droplet infiltration rate and the shorter infiltration time. 

#### 2.3.4. Process Optimization

At present, many researchers focus on the realization stage of the forming process, that is, the successful processing of a certain material is realized by using BJ technology. Besides, some researches of process mechanism and optimization are carried to tackle the issue of parts strength and accuracy. Reasonable post-processing is essential to achieve the required density, strength, and precision of the part. Currently, the density and strength are ensured by process of low temperature pre-curing, isostatic pressing, sintering, and infiltration, and so on. The accuracy is improved by powder removal, grinding, and polishing process [58,59]. On the basis of the existing post-processing technology, the development of new processes is an urgent task to improve the quality of the parts.

For process optimization, considerable attention has been paid to investigate how process parameters affect the quality produced by BJ, such as printing speed, layer thickness, drying time. Miyanaji et al. [60] studied the influence of printing speed on the dimensional accuracy of printed parts, and concluded that increasing the printing speed would affect the printing accuracy. Chen et al. [61] concluded layer thickness is the main factor that affects the surface roughness and drying time has the most significant effect on the mean shrinkage rate along Y-axis and Z-axis. Besides, simulation is another effective solution to do process optimization. Bai et al. [62] proposed a new approach based on sessile drop goniometry on a powder substrate to study the binder-powder interactions aiming to optimize the printing process.

## 3. Selective Laser Sintering (SLS)

### 3.1. Overview 

**Figure 6:**
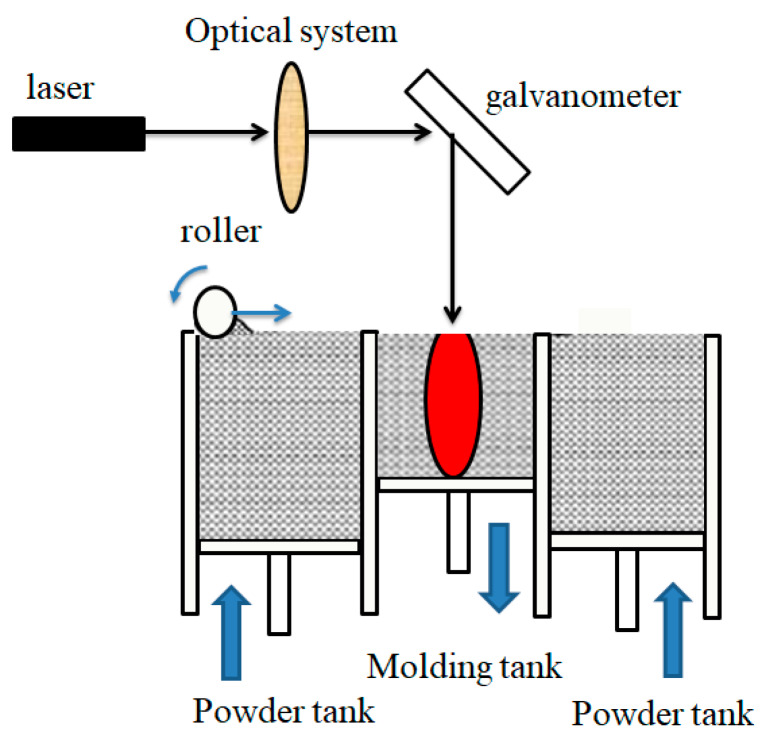
Working scheme of SLS.

Selective laser sintering (SLS) also referred to as laser sintering, it is a technology which melts the powder materials directly by laser energy to construct three-dimensional parts. Polyamides (PA6, PA11, and PA12) based materials, single or composite, are the main materials for SLS. PEEK, polypropylene and some elastomer materials such as TPU are also available now [63]. CO_2_ lasers are commonly used in the SLS process to provide sufficient energy [64,65]. The working process is as follows: first, the model file is converted into STL format and imported into the computer. Then, the computer controls the laser to scan the profile according to the cross-section data, selectively sinters the powder and completes a layer of printing. Finally, the roller lays a new layer of powder and the laser scans the new layer again. Layer stacking cycles back and forth until the 3D part is fully formed [66]. After cooling and removing excess powder, the designed parts can be obtained. The working scheme is shown in Figure 6.

**Figure 7:**
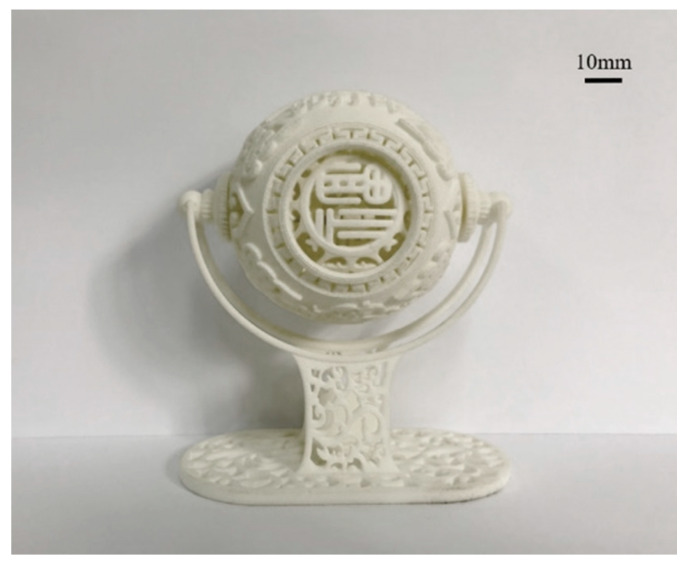
SLS printed artwork.

By analyzing the working scheme of SLS, technical characteristics can be summarized. First of all, powder used in this technology can be wide, although the fact is Polyamide 12 remains by far the most widely used laser sintering polymer. This is because the main process of SLS involves preheating the powders from room temperature to above the melting point to allow sintering. The thermal behaviors of different polymers can make shrinkage and distortion more likely. But in theory, most powder based materials can be used in SLS process as long as exposing to high enough laser power and optimum wavelength for energy absorption, so it has the potential to develop a wide range of materials. Second, it is suitable for the production of small batch and customized parts; its processability is relative ease as well within the same mechanical performance. Tensile strength and Elastic modulus of laser sintered polyamides are now comparable to their injection molded counterparts. Third, SLS technology has high accuracy. When the particle size of powder material is less than 0.1 mm, the accuracy can reach ±1%. Finally, because of its diversified materials selection, SLS technology can be applied to many industries such as automobile, aerospace, medical, artware etc. A typical part produced by SLS is shown in Figure 7. However, the use of laser increases the operating cost of the machine, and the scanning speed of the laser affects the printing speed as well.

### 3.2. The Development of SLS

SLS technology was first proposed by C.R.Dechard of the University of Texas Austin in 1989 in his master’s thesis and got patent in 1990 [67,68], and then he founded DTM and brought this technology to the market. In 1992, DTM unveiled the commercial rapid prototyping machine named Sinter station to the market. EOS, a German company founded in 1989, focuses on high-end SLS technology, launched EOSINT series laser sintering machines after DTM [69]. In 2001, SLS was promoted by 3D Systems and gradually developed into one of the most reliable 3D printing technologies [70]. In September 2011, Hunan Farsoon High-Technology company in China successfully developed SLS equipment FS401, becoming the third manufacturer of high-end SLS equipment in the world. The HRPS equipment of Hua Zhong University of science and technology in China used the dynamic focusing system of vibration-mirror, and the volume can be reached 1400 × 1400 × 500 mm^3^, ranking the leading position of the world. EOS launched a P810 system specially designed to deal with HT-23 materials in the aerospace industry in 2018, with a construction volume of 700 × 380 × 380 mm^3^, two lasers, and superior performance of the formed parts. HT-23 is also the first type of high-carbon fiber PEEK material that can be processed on an EOS system. 3D Systems and EOS are currently the two largest suppliers of the SLS equipment.

### 3.3. The Development of SLS

At present, the SLS research mainly focuses on process optimization, material development, and application extension.

#### 3.3.1. Process Optimization

With the development and progress of society, the accuracy, quality, and speed of printing are increasingly important. Printing process as the main procedure in SLS has attracted more attention both in commercial and research field, the optimization of process is recognized as a crucial solution to deal with these problems.

Laser power and scanning speed are the main parameters affecting the sintering process. Laser power shows the photon energy per unit time being irradiated on the selective sintering area, scanning speed influences the time duration for the target area. These two factors determine the total energy being absorbed by polymer powders which in turn affects the quality and accuracy of the final part. Solid, stronger parts were fabricated under high energy density level while porous, weak parts were produced using low energy density levels, different types of lasers and laser parameters and their effects on SLS can be found in Lee et al. [71,72]. Singh et al. [73] studied dimensional accuracy of SLS considering the effect of different process parameters. It was concluded that dimensional accuracy increases with the increase in laser power, bed temperature, and scan count, while decreases with the increase in scan spacing. Bai et al. [74] studied the influence of processing conditions on the mechanical performance of printing parts, and concluded that reducing the cooling rate could improve the performance of printing parts. Li et al. [75] obtained the effect of laser power on the density and tensile strength of the part, followed by the effect of layer thickness, preheating temperature, and scanning speed. Besides, some simulations and modeling were also carried out to investigate the real printing process and optimize the parameters. Mokrane et al. [76] addressed modeling, simulation, and validation aspects that are indispensable for studying and optimizing SLS process, part shrinkage and layers deposition were involved. Dastjerdi et al. [77] used a genetic algorithm to solve the minimum warping process parameters of PA12 powder under constant energy density, and proved that the finite element model is an appropriate method to simulate sintering process and can guide the corresponding parameter optimization.

#### 3.3.2. Printing Material 

Polymer materials are the earliest applied SLS materials because of their mild forming conditions, special physical and chemical properties, and high product accuracy. However, the existing polymer materials are limited, in which nylon materials account for over 90%. Polymer composites are flourishing to enhance the properties of the laser sintered parts since pure polymer properties are limited, additives and reinforcements should be added to the base material for strengthening and functionality purposes [78,79]. Bai et al. [80] applied solvent precipitation method to evenly coat carbon nanotubes with mass fraction of 0.1% on the surface of nylon 12 particles, effectively improving the thermal conductivity and mechanical properties of SLS nylon 12 composite. Salmoria et al. [81] investigated the structure and properties of PA12 and PBT blend composites produced by SLS and verified the potential application in the manufacturing of blend parts with a composition of PA12/PBT with high stiffness and fatigue resistance. As a kind of high performance polymer, PEEK and its composites become the promising candidates that satisfy the demands for high stiff and lightweight. However, the strength of the PEEK composites manufactured by SLS is lower than their injection molded part. So Yan et al. [82] added carbon fibers to PEEK to reinforce the strength; the experimental results showed high tensile strength and elasticity modulus can be achieved. Besides, new printing material and solution should be come up to improve their productivity and processibility.

#### 3.3.3. Application Extension

**Figure 8:**
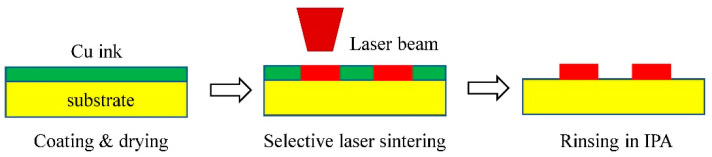
SLS printed micro PCB (IPA: isopropyl alcohol).

SLS technique offers a novel strategy to achieve the integration of printing material, which has been successfully applied in many fields, such as automobile, aircraft, spacecraft, art ware. With an emerging group of additives were proposed, new fields are constantly being expanded [83]. Min et al. [84] changed the powder bed in SLS technology to the substrate coated with photosensitive ink, and successfully printed the micro PCB, broadening the SLS to the electronic field, as shown in Figure 8. At the same time, SLS has a broad application prospect in the field of biological medicine, especially in tissue engineering and medical implantation. For example, Eosoly et al. [85] produced TE scaffold by SLS technology using PCL/Hap composites as input materials. The mechanical properties of the scaffold are stable and the cellular metabolic activity is high.

## 4. High-Speed Sintering (HSS)

### 4.1. Overview

**Figure 9:**
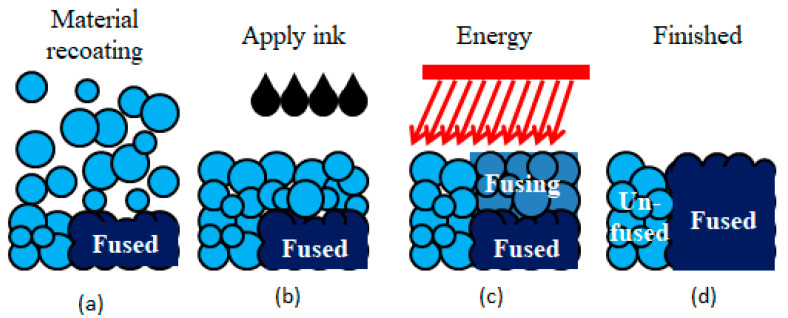
Mechanism of HSS. (**a**) Material recoating; (**b**) applying ink to the selected area; (**c**) irradiation by infra-red lamp; (**d**) powders with ink are fused.

HSS is the abbreviation of “high-speed sintering,” and its main materials are nylon and a few elastomer materials. In the HSS process, the nozzle selectively deposits a radiation absorbing material (RAM) directly on to the polymer powder surface according to the cross-section data, and then, followed by irradiation by an infra-red lamp to raise the temperature of the powder in a short time, this creates a substantial difference between the IR absorptivity of the areas deposited with RAM and those without. Areas which have been deposited with RAM absorb sufficient energy to sinter the powder particles. This process then repeats until the build is completed [86], the working process is shown in Figure 9.

HSS is a novel rapid manufacturing technology, which has the following characteristics. First, the printing process only needs RAM as the printing additives, and replaces the laser with an infrared lamp, which greatly reduces the cost of the device. Second, the printing speed of HSS is greatly improved compared to SLS. The current SLS technology fuses material powders with a laser scanning, which restricts the productivity to some extent; HSS uses infrared lamps to heat a large printing area, which increases the rate of sintering in each layer, making it possible at speeds and cost as that of the rival injection molding. Third, the recycling rate of the powder is high. HSS will not reduce the surface quality of the printing parts and the processing does not require a nitrogen-protected environment.

### 4.2. The Development of HSS

**Figure 10:**
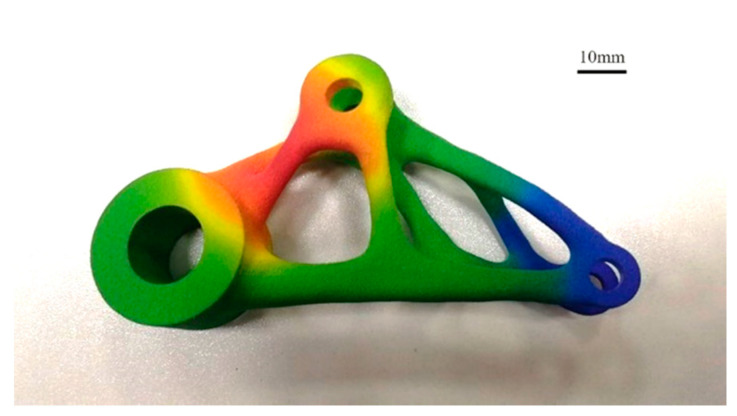
HP printed color part.

HSS technology was first proposed and patented by Professor Neil Hopkinson at Loughborough University in 2003 [87], and then Hopkinson has been working on this technology ever since. In 2013, the first commercial HSS printer was installed in the University of Sheffield. The German Voxeljet company, which is mainly devoted to the printing, also launched a new HSS printer, VX200 in 2017 for trial-manufacture of functional prototypes and production of terminal parts. At the same time, HP also launched its Multijet Fusion technology. This technology applies detailing agents based on HSS; the detailing agents are jetted on the edge of the melting area to ensure the edges are smooth and accurate. In 2016, HP launched MJF 3D 4200/3200 printer based on HSS. The effective construction volume of the equipment reached 380 × 284 × 380 mm^3^, the printing speed reached 4000 cm^3^/h, and the layer thickness reached 0.08mm. Based on internal testing and simulation, considering the number of parts printed at the same time, HP printing speed is ten times higher than traditional SLS, and the average printing cost is half of that of similar products [88]. In 2018, HP launched the MJF full-color printer 3D580, which has a wider application area, the printed part is shown in Figure 10. In 2019, Wittkopf et al. [89] at HP’s lab developed a new approach to build conductive traces, vias and contacts on a printed part used for electronic field. In the same year, Voxeljet released a new technology—greyscale 3D printing—based on HSS process. This technology can create singular parts with variable materials properties by controlling the ink amount jetted by the nozzle.

Comparing HSS and MJF, we can find that the two technologies are very similar in principle. Both involve spraying the RAM on the surface of the powder bed but MJF applies detailing agents to ensure edge fitness of printing object.

### 4.3. Research Focus and Prospects of HSS

As big companies, such as Voxeljet, start to commercialize the HSS technology, more attention has been paid in the research and development of HSS recently. The research hotspots mainly focus on process, device, and materials development.

#### 4.3.1. Parameter Optimization

The printing parameters in HSS mainly include the ink density, powder bed temperature, and the power of the infrared lamp, and so on. It is very important to study the influence of these parameters on the sintering parts to guide the process improvement. Hopkinson [90] studied the effects on powder bed hardness with powder bed temperature and infrared lamp power, which proved that the powder bed hardness will increase with the increase of powder bed temperature and infrared lamp power. Therefore, it is necessary to balance the relation of the powder bed temperature and the power of the infrared lamp to achieve the result of easy post-processing. Majewski [91,92] used HSS devices to print parts and compare the mechanical performance of the parts under different parameters, which proved that the increase of infrared lamp power and preheating temperature within a certain range would improve the mechanical performance of the parts. Ellis et al. [93] studied the effect of ink density on the microstructure and mechanical properties of nylon parts. It was concluded that as the density of ink increased, the stiffness and tensile strength of nylon parts increased, but the ductility decreased. The addition of ink had little effect on the microstructure of nylon powder after sintering. Garrett et al. [94] considered impact of extended sintering times on mechanical properties in PA12 parts produced by powder bed fusion processes and found slower heating methods like HSS and MJF produce large elongation, cooling rate also appears to play a significant role. Further work is needed to study more closely the impacts of cooling rates on the mechanical properties to optimize the performance.

#### 4.3.2. Development of New Materials

As a novel 3D printing technology, high-speed sintering (HSS) widely uses Nylon-12 and a few elastomer materials as the standard material currently. Many scholars hope to broaden the kinds of materials in this field, for example, Hopkinson et al. [95] selected two commercially available laser sintering grade powder, HST10 and ALM TPE 210-S. Tensile test specimens were manufactured using each material and mechanical properties analyzed and compared to the manufactures’ specification for laser sintering. The results indicate that HSS is capable of processing laser sintering grade polymeric powders and may extend beyond. In 2018, Voxeljet has released two new HSS printing materials—PP (Polypropylene) and TPU (Thermoplastic Urethane), which are already being tested in the automotive and sports manufacturing industry. From this point of view, the development of printing materials will continue to be a research hotspot in this field.

Moreover, as an important material in HSS technology, RAM determines the printing speed, the quality, and accuracy of the final part to a large extent, so it is very important to develop RAM which matches with the printing materials. RAM should meet the characteristics of rapid absorption of infrared energy, wide viscosity range, and large temperature range. Besides, realizing color printing by developing RAM is also a new trend of HSS development.

#### 4.3.3. Commercialization of HSS

Because of the short development time of HSS technology, there are few HSS commercialized devices. At present, the main companies developing commercial HSS equipment are Voxeljet and HP, HP with its excellent printing speed and performance occupies a leading position in the market. HSS technology has obvious advantages, and it is sure that the commercialization process will accelerate.

## 5. BJ and SLS and HSS Technology Comparison

BJ, SLS, and HSS are three important technologies for 3D printing of polymer powder materials. With the continuous maturity and development of AM, there are increasing printing systems showing in the market. 3D Systems, EOS, HP, Farsoon et al. which dominate most of the market share have launched a variety of devices used in different field and industries, a summary of commercial 3D printing machines and some official parameters are shown in Table 1.

Additionally, SLS and HSS are both heat-based technologies, a comparison between their sample’s properties is necessarily shown in Table 2.

Through the comparisons above, it can be found that MJF has some advantages in dimensional accuracy, surface roughness, and the printing speed which will greatly improve the efficiency for industries. Vertical dimensional accuracy of the SLS parts is not as good as MJF, which might be due to the temperature gradient caused by high energy input in a very short time. The printing accuracy of BJ is the highest among the three technologies, which comes from the high-precision nozzles in the print head. Surface roughness of MJF is improved by applying detailing agent. MJF uses infrared light with a large heating area as a heat source, shortening the fusing time of each layer powder which significantly increases the printing speed. However, relative lower infrared energy of MJF causes the lower density of the printed part. To improve the printing speed, multi lasers are applied in some SLS machines, shown in Table 1. In the meantime, the properties comparison between MJF and SLS are mix from the literature, which may come from non-standardized processing procedures from different machines and users. For example, the mechanical properties of PA12 processed by MJF was a little lower than SLS studied by Xu et.al [101] (Table 2), however, Sillani et al. [102] found the difference that tensile strength and elongation at break of MJF samples were better than SLS while Young’s modulus was lower. Generally, the mechanical performance for the MJF and SLS parts are in the same level, which are in the upper-class of polymer 3D printing techniques. For binder jetting process, the parts are formed by physical bonding, and the porosity is generally high, so the mechanical property is the worst among three, only about 10–30 MPa [96].

For materials shown in Table 1, until now, powder materials used in the market are mainly some industrial polymers for manufacturing applications such as PA, PEEK, PS. For continuous developments and applications, there is an imperative need for developing advanced polymers and composites that can be applied in different types of fields. In fact, a benefit of BJ and SLS is that there are no specific limitations of the materials selection as long as matched binders and lasers are used. For HSS, RAM should be given more attention because it is greatly related to the success of the printing.

As applications of additive manufacturing has been increasingly extensive, appropriate process selections are more important for time saving and waste reduction. According to the above comparisons and characteristics of each technology, suggestions of process selections are given as follows: Binder jetting technology are suitable for concept modeling and rapid prototyping because of the multi-color printing functions, high dimensional accuracy, and low cost. Selective laser sintering, as the most mature technology, can be applied in aerospace, automobile, and biomedical applications with more materials choices and good performance. However, printing speed of the SLS process may be a limited factor for large quantity fabrications. High-speed sintering, with its comprehensive performance of printing speed and mechanical property, should be widely used in large-scale terminal-part production and manufacturing industries. Nevertheless, the maintenance cost of HSS seems higher compared to the other two technologies, but as the technology develops, the maintenance cost and price of the whole machine will definitely decrease.

## 6. Summary and Outlook

High powder utilization, free of support structure, great design freedom, easy manufacturing process, and extensive application fields especially in biological systems are the main benefits of polymer powder 3D printing technologies. A comprehensive review of polymer powder printing methods including binder jetting technology, selective laser sintering, and high-speed sintering were carried out. This article reviewed introduction, development, research focus, and prospects of each technology respectively. The comparisons and process selections were also discussed.

Binder jetting technology, which has the longest history among these three techniques, is especially popular for the fabrication of 3D multicolored models. However, the final part produced by this process is weak and porous because of the physical bonding between layers. Thus, process optimizations not only involve the printing procedure but also the post-processing process that are necessary to improve the property performance. Printing materials and binders have still been a research point. Additionally, printhead as the core hardware of the mechanical structures needs further development to meet different requirements in different binders. Selective laser sintering, which has been considered as the most reliable 3D printing technology, has been widely attracted by manufacturing industries because of the high mechanical performances and mature processing chain. However, the existing polymers are limited and thus the resulting properties are restricted as well. Different additives and reinforcements are added to the base materials to enhance the properties of laser sintered part. Printing procedure as the main part in SLS has also gained attention both in commercial and research field, studying the relations between printing parameters and final part quality has been popular as well. The application in SLS has been extended these years, electronic and biological field were included. High-speed sintering, as a novel 3D printing technology, has great potential in AM industries, further research of forming mechanism, printing simulation, materials development should be carried out.

As a novel approach, additive manufacturing provides a promising manufacturing for engineering and advanced functional materials. Polymer powder-based 3D printing technologies have been rapidly developing since its inception. In reality, polymer powder-based AM has complemented traditional manufacturing in some areas such as concept modeling and rapid prototyping. However, key issues such as the number of materials available, the cost of AM machines and materials, building time, mechanical properties, and accuracy of the final printed parts are still big hurdles and challenges for successful development of AM technologies. Currently, only a few polymer powder materials can be processed to meet the required quality specifications, and standardization is necessary for those that can. The reason is either the raw powders of materials are difficult to produce or materials cannot meet the processing requirements, for example, suitable thermal transforming interval for SLS and HSS. The cost of AM machines and materials are still relatively high for small-scale production. Some machines can cost tens or even millions of dollars. Even manufactures have access to the equipment, the building time may limit the production efficiency and increase the cost. Although a big advantage of powders is recyclability, i.e., the use of excessive powders for next printing, those polymer powders recycled may suffer from a quality loss. Besides, mechanical properties, surface finish, and accuracy of the parts should be more stable and controllable in one printing circle.

To tackle these challenges, there are some expectations and outlooks. First, new polymer and its composites need further development, especially industrial polymers for manufacturing applications and biodegradable polymers for biomedical field. Meanwhile, development of globally defined standardizations of materials must be a top priority. Second, the rise to prominence of polymer powder-based AM has influenced the manufacturing and quality of the raw polymer powders, and more convenient and economical polymer powder fabrication methods could be investigated. Third, post-processing procedures can be eliminated or reduced, which is essential for improving the competitiveness and decreasing the cost, especially some intensive manual operations that are dangerous for AM parts. Then, in terms of the property issues, pores existence and property anisotropy should be avoided, both of which are highly detrimental to the mechanical strength and surface finish. Since AM is a layer upon layer process, parts with graded compositions and structures is a promising direction for multifunctional applications. Finally, multicolor 3D printers have emerged in the past few years and will become increasingly popular in the near future.

With the continuous development of AM, commercial devices will become more refined, multifunctional, and generally affordable. As more and more countries invest in research of process, equipment, materials, and related studies, polymer powder 3D printing will play a more important role in various fields.

## Figures and Tables

**Table 1:** Commercial devices of polymer powder based technology.

Technology	Machine *	Build Space * (L × W × H) (mm^3^)	Layer Thickness * (mm)	Build Rate *	Materials *	Properties *	Others *
Binder Jetting	ProJet CJP 860pro (3D Systems)	508 × 381 × 229	0.1	5–15 mm/h	VisiJet PXL	Dimensional accuracy: 0.15 mm; Tensile strength: 10–30 MPa	Full color
	ProJet 360 (3D Systems)	203 × 254 × 203	0.1	20 mm/h	VisiJet PXL		White (single color)
Selective Laser Sintering	HT1001P (Farsoon)	1000 × 500 × 450	0.06–0.3	15 L/h	PA6 PA12	Density (sintered part): 0.9–0.95 g/cm^3^; Tensile modulus(x,y)/(z): 1.7/1.65 Gpa; Tensile strength(x,y)/(z): 48/47 MPa; Elongation at break(x,y)/(z): 20/10%	2*CO_2_ Laser (100 W)
	P760 (EOS)	700 × 380 × 580	0.06/0.1 /0.12/0.15/0.18	32 mm/h	PA12, PA12 composite (aluminite powder/carbon fiber/fiberglass PA11/ Polystyrene		2*CO_2_ Laser (50 W)
	P110 (EOS)	200 × 250 × 330	0.06/0.1 /0.12	20 mm/h			CO_2_ Laser (30 W)
	sPro230 (3D Systems)	550 × 550 × 750	0.08–0.15	3 L/h	DuraForm PA/GF/EX/HST/Flex/PS		CO_2_ Laser (70 W)
High Speed Sintering	MJF3D4200 (HP)	380 × 284 × 380	0.07–0.1	4.115 L/h	PA12/PA11	Dimensional accuracy: ±0.3%	Infrared energy as heat source
	VX200 (Voxeljet)	300 × 200 × 150	0.08–0.1	No specific data	PA12/TPU	No specific data	

* All data are from official data sheets [88,96,97,98,99,100].

**Table 2:** Property comparison between SLS and HSS (PA12) [101].

Items	SLS	MJF
Surface roughness (bottom) (μm)	14.40 ± 1.06	6.31 ± 0.43
Printing speed (mm^2^/s)	1250	10792
Crystallinity (%)	24.37	30
Dimensional accuracy (vertical) (mm)	±0.25	±0.1
Density (g/cm^3^)	0.99 ± 0.04	0.93 ± 0.08
Tensile strength (MPa)	43.61 ± 0.46	40.10 ± 1.49
Elongation at Break (%)	31.55 ± 2.93	17.45 ± 3.87
Young’s Modulus (GPa)	1.76 ± 0.02	1.42 ± 0.04
